# The Medicaments of Brutes

**Published:** 1883-08

**Authors:** 


### THE MEDICAMENTS OF BRUTES.

In a communication to the Biological Society of London, recently sent by M. Delaunay, on the medical practice of animals, the doctor gave some interesting facts, from which he argued that the human reason ought to be trusted as much as animal instinct, in many instances where medical science seems to be at fault; and he insists that the desire of sick persons for certain foods and drinks may be a natural instinct rather than a morbid fancy.

But he does not state how the one may not be mistaken for the other. In his list of examples of medical instinct in the lower animals, M. Delaunay says that animals bathe for cleanliness and health, that they get rid of their parasites by using dust, mud, clay, etc. Those suffering from fever restrict their diet, keep quiet, seek darkness and airy places, drink water, and sometimes plunge into it.

When a dog has lost his appetite, he eats that species of grass known as dog's grass (*dogtooth*), which acts as an emetic and purgative. Cats also eat grass. Sheep and cows, when ill, seek out certain herbs. An animal suffering from chronic rheumatism always keeps, as far as possible, in the sun. If a chimpanzee be wounded, it stops the bleeding by placing its hand on the wound, or dressing it with leaves and grass. When an animal has a wounded leg or arm hanging on, it completes the amputation by means of its teeth. A dog, on being stung in the muzzle by a viper, was observed to plunge its head repeatedly for several days into running water. The animal eventually recovered.

A sporting dog was run over by a carriage; during three weeks in winter it remained lying in a brook, where its feed was taken it. The animal eventually recovered. A terrier hurt its right eye; it remained lying under a counter, avoiding light and heat, although it habitually kept close to the fire. It adopted a general treatment, rest and abstinence from food.

The local treatment consisted in licking the upper surface of the paw, which it applied to the wounded eye, again licking the paw when it became dry.

The doctor thinks that veterinary medicine, and perhaps human medicine, can gather from these facts useful indications, precisely because they are prompted by instinct.

